# Weighted Least-Squares Finite Element Method for Cardiac Blood Flow Simulation with Echocardiographic Data

**DOI:** 10.1155/2012/371315

**Published:** 2012-01-16

**Authors:** Fei Wei, John Westerdale, Eileen M. McMahon, Marek Belohlavek, Jeffrey J. Heys

**Affiliations:** ^1^Chemical and Biological Engineering Department, Montana State University, Bozeman, MT 59717, USA; ^2^Mayo Clinic, Scottsdale, AZ 85259, USA

## Abstract

As both fluid flow measurement techniques and computer simulation methods continue to improve, there is a growing need for numerical simulation approaches that can assimilate experimental data into the simulation in a flexible and mathematically consistent manner. The problem of interest here is the simulation of blood flow in the left ventricle with the assimilation of experimental data provided by ultrasound imaging of microbubbles in the blood. The weighted least-squares finite element method is used because it allows data to be assimilated in a very flexible manner so that accurate measurements are more closely matched with the numerical solution than less accurate data. This approach is applied to two different test problems: a flexible flap that is displaced by a jet of fluid and blood flow in the porcine left ventricle. By adjusting how closely the simulation matches the experimental data, one can observe potential inaccuracies in the model because the simulation without experimental data differs significantly from the simulation with the data. Additionally, the assimilation of experimental data can help the simulation capture certain small effects that are present in the experiment, but not modeled directly in the simulation.

## 1. Introduction

The physics of blood flow in the left ventricle of the heart has traditionally been studied using either experimental measurement of flow properties (e.g., ultrasound or magnetic resonance imaging) or computational fluid dynamic models. Experimental approaches are generally limited to obtaining flow information at only few spatial locations and using time-averaged properties. Computational models require assumptions along the mathematical domain boundaries, and they include numerical approximation error and model error. In many cases, however, it is desirable to have the more comprehensive spatial and temporal data provided by computational fluid dynamics combined with the data provided by experimental measurement. The weighted least-square finite element method (WLSFEM) is a computational modeling approach that allows experimental data to be assimilated into the model in a flexible framework so that the numerical approximation matches the more accurate experimental data while, at the same time, not being contaminated by errors in the noisier experimental data. The application of this method to the simulation of blood flow in the left ventricle is examined here.

One approach that we have used previously for obtaining experimental blood flow data in the left ventricle is echocardiographic particle imaging velocimetry (echo PIV) [1–7]. For this approach, microbubbles are introduced into the blood, and they are imaged using 2D brightness (B)-mode ultrasound scans. These images are acquired at a rate of approximately 60 frames/sec, and the microbubble concentration is kept low enough that individual bubbles can be tracked between frames. Using cross-correlation analysis, the particle displacement between two images in sequence can be calculated, and, after dividing by the time span between images, PIV software can calculate the two velocity components tangential to the imaging plane. Echo PIV has been used as a research tool for nearly a decade, and it has been validated by multiple research groups [1, 7]. The flow velocity data provided by echo PIV is useful, but there is a strong interest in using the data to determine addition flow property information such as pressure gradients and viscous energy loses. The problem is that the calculation of these, or almost any other additional flow property, requires full 3-dimensional velocity data. The data from echo PIV are limited to a single 2D plane. As a result, 2D ultrasound scans provide only 2 components of a 3D velocity field.

A complementary approach to echo PIV that could allow the approximation of all 3 components of the 3D velocity field is to use computational fluid dynamics to simulate blood flow in the left ventricle. A number of computational models have been developed specifically to model blood flow in the heart (c.f., [8, 9] and references therein). Many of these models also predict the motion of the heart wall and simulate the blood-tissue interaction (e.g., [10–12]). The enforcement of boundary conditions with most computational fluid dynamics approaches, such as the finite element or finite volume methods, is achieved by strongly enforcing the velocity (i.e., exactly matching the experimental velocity data) at nodes within the discretization mesh or grid [13, 14]. There are two problems with strong enforcement of the experimental data at the nodes: (1) the experimental data might not be known at the exact location of the nodal mesh points so some type of interpolation is required, and (2) the errors in the experimental data will be propagated throughout the 3D domain and contaminate the numerical approximation at all nodes throughout the computational domain [15]. What is needed is a computational fluid dynamics approach that can assimilate velocity data anywhere in the domain, not just at computational nodes, and an approach that can weakly enforce the experimental data with a weighting that is varied depending upon the accuracy of the experimental data.

In an earlier paper, we developed the WLSFEM for the assimilation of data when solving partial differential equations, including the steady Navier-Stokes equations [15]. This paper extends that work in a number of different ways so that the method can be applied to the simulation of blood flow in the left ventricle using echo PIV data. The greatest change is caused by the fact that we now have a moving fluid domain so a pseudosolid domain mapping technique is developed to handle the deforming fluid mesh. The mathematical approach that we have developed and the numerical implementation are described in the next section. This approach is then applied to two different example problems: a moving flap problem and blood flow in the left ventricle.

## 2. Methods

The physical phenomena of interest here are typically modeled by partial differential equations. In particular, incompressible, Newtonian fluids are modeled by the Navier-Stokes equations, which are generally considered appropriate for modeling blood flow in the heart [16, 17] and are given by:

(1) Re(∂v∂t+v·∇v)=−∇p+1Re∇2v,∇·v=0,

where **v** is the dimensionless velocity, *p* is the dimensionless pressure, and *Re* is the Reynolds number. It should be noted that the Reynolds number is not written only on the viscous or convective terms, as it normally is, but the equation is instead scaled so that the Reynolds number appears on both terms. The Navier-Stokes equation is generally considered appropriate for modeling blood flow in the heart [16, 17]. The WLSFEM begins by defining new variables so that all second-order equations can be rewritten as systems of first-order equations. There are a number of different options available for rewriting the Navier-Stokes equations as a first-order system, and this choice can significantly impact the properties of the resulting discrete approximation [18–20]. For the particular problems of interest here, accurate mass conservation is of importance, so a modified vorticity approach is used [21]. This approach begins by defining a new variable, called the vorticity, by

(2) ω=−∇×v,

and another new variable, **r**, by

(3) r=∇p+Re2∇|v|2=∇(Re2|v|2+p),

which is often referred to as the gradient of the total pressure. Using these two new variables, the Navier-Stokes equations (1) can be rewritten as the following system of first-order equations:

(4) ∇×v+ω=0,∇·v=0,1Re∇×ω−r−Re(v×ω+∂v∂t)=0,∇·ω=0,∇×r=0,∇·r−Re(ω·ω)−Re(v·r)=0.

In the least-squares finite element method, this system of equations is cast as an optimization problem based on the functional:

(5) G(v,ω,r)=||∇×v+ω||0,Ω2+||∇·v||0,Ω2+  ||1Re∇×ω−r−Re(v×ω+∂v∂t)||0,Ω2+||∇·ω||0,Ω2+||∇×r||0,Ω2+||∇·r−Re(ω·ω)−Re(v·r)||0,Ω2+wΓh||v−g1||0,Γ2+1h||ω−g2||0,Γ2+wPIVh||v−gPIV||0,ΓPIV2,

where ||·||_0,*Ω*_^2^ is the L^2^-norm on the 3D fluid domain and (*w*/*h*)  ||·||_0,Γ_^2^ is the weighted L^2^-norm along the 2D boundary surfaces (Γ) or 2D surfaces, where PIV data is given (Γ_PIV_). The PIV plane is simply a 2-dimensional cross-section that is typically somewhat near the middle of the 3-dimensional domain (Γ_PIV_), where as the other boundaries (Γ) are all on the surface of the 3-dimensional domain. The weighted L^2^-norm, used along the boundary surfaces, is an approximation of the H^1/2^-norm that deemphasizes oscillatory components (i.e., noisy components) relative to the H^1/2^-norm [22]. The functions *g*_1_, *g*_2_, and *g*_PIV_ are the given boundary or PIV data, that is, to be weakly matched by the numerical approximation of the solution. For example, *g*_1_ is set to the surface displacement rate along no-slip boundaries. The function *g*_2_ is only set along boundaries where the normal vorticity is known, such as along walls. It is also straightforward to enforce this data strongly on the finite element space so that it is matched exactly by the approximate solution, but that is not an optimal strategy if the data contains errors, which is generally true for experimental data. Finally, the PIV data, *g*_PIV_, can be either 2- or 3-dimensional data, but the PIV method is typically limited to providing 2-dimensional data, so that is the focus here. The spatial location of the PIV data does not need to be the same as the computational mesh node locations. The data can be located anywhere within the computational domain.

The boundary functional weights, *w*_Γ_ and *w*_PIV_, should be chosen so that the weight value is larger in regions where the given data, *g*, is known more accurately and smaller in the regions where the data contains more noise. Along the heart walls, for example, we know that the fluid velocity is equal to the velocity of the wall, but the wall location is not known precisely so there is still some error. In the problems of interest here, the PIV data typically contains larger errors than the boundary data, so we would expect *w*_Γ_ > *w*_PIV_, which would result in an approximate solution that more closely matches the boundary data than the PIV data. In [15] it was shown that the boundary functional weight should be chosen by

(6) $$w\approx \frac{1}{\sigma^{2}},$$

where *σ* is the standard deviation in the given data. To simplify this process, we typically set the boundary functional weight to 1.0 for the most accurate boundary data, and then the other boundary functional weights are set relative to the most accurate data. The vorticity is typically determined from the velocity data, and boundary conditions on the vorticity are weighted consistently with the accuracy of the velocity data.

When modeling blood flow in the left ventricle, or any fluid-structure interaction problem, the shape of the fluid domain is continuously changing. There are a number of numerical strategies for addressing the changing domain shape, including the generation of a new mesh every time step or grid mapping using equations such as the Winslow generator [23, 24]. Another straightforward method is to solve a compressible elasticity problem over the fluid domain and use the solution from the elasticity problem to move the nodes of the finite element mesh. This approach is often referred to as a pseudosolid domain mapping technique [25, 26]. The linear, compressible elasticity equation can be written as

(7) $$\lambda \nabla \left(\nabla \cdot u\right)+\nabla^{2}u=0,$$

where **u** is the displacement and *λ*, which is typically set to 1.0, is a Lamé coefficient related to Poisson's ratio. Similar to the Navier-Stokes equation, this equation also must be rewritten as a first-order system of equations by defining a matrix of new variables, *U*, equal to the gradient of **u**. The full first-order system is

(8) $$\begin{array}{c} U-\nabla u=0,\\ \lambda \nabla \left(\mathrm{tr}\left(U\right)\right)+\nabla \cdot U\\ \nabla \times U=0, \end{array}n=0,$$

where tr⁡(*U*) is the trace of *U*. The equations in the first-order system are combined into the functional

(9) $$\begin{array}{cc} G_{u}\left(u,U\right)= & {||U-\nabla u||}_{0,\Omega }^{2}+{||\lambda \nabla \left(\mathrm{tr}\left(U\right)\right)+\nabla \cdot U||}_{0,\Omega }^{2}\\ & +{||\nabla \times U||}_{0,\Omega }^{2}+{\frac{1}{h}||u-g_{u}||}_{0,\Gamma }^{2}, \end{array}$$

where *g*_*u*_ is the given boundary displacement. It is important to note that moving the finite element mesh can create an additional, artificial convection that must be subtracted from the actual convective velocity in the Navier-Stokes equation [25].

The WLSFEM has a number of computational and algorithmic advantages for the problem of solving the Navier-Stokes equations and pseudosolid domain mapping equations with assimilated data:

- it provides tremendous flexibility in handling the additional conditions imposed by the experimental data, including the ability to weight data based on the accuracy of the experimental data, that is, accurate data can be weighted and matched more closely by the CFD solution while less accurate data is only loosely matched by the CFD approximation;
- the mathematical framework of least-squares minimization leads to symmetric positive definite matrices, which generally allows for efficient algebraic multigrid solvers [27];
- the functional itself provides a natural sharp local error estimator, which could enable effective adaptive refinement [28, 29].

To solve the least-squares problem, the equations in the functional (*G*) are first linearized so that the solution can be found using a Gauss-Newton approach. The least-squares weak form is converted into a linear system of equations by choosing a finite element basis. All the results presented here utilized a triquadratic finite element basis. The WLSFEM allows the solution spaces for the variables to be chosen independently, and there is no restrictive stability condition (i.e., inf-sup condition) to satisfy [30]. As a result, all variables in the reformulation of the Navier-Stokes equations or the linear elasticity equation can be approximated with the same basis.

All simulations were performed using the ParaFOS code, written by the authors. The code imports hexahedral meshes from the Cubit mesh generation package (Sandia National Laboratory). The finite element meshes are then partitioned using the Metis graph partitioning library [31]. The software is designed to run on distributed memory clusters using the MPI library for communication. The linear matrix problem generated during each Gauss-Newton step is solved using the hypre library of solvers (from the Center for Applied Scientific Computing, Lawrence Livermore National Laboratory, see [32]). Specifically, the BoomerAMG parallel algebraic multigrid solver is used as a preconditioner for a conjugate gradient iteration.

## 3. Results

To test the WLSFEM on problems with moving domains and PIV data assimilation, two different test problems are examined. The first problem is a flexible flap that is displaced by a fluid jet, and optical PIV is used to obtain experimental data. The second problem is a simulation of blood flow in the left ventricle of a pig using echo PIV data. It is this second problem that was the primary motivation for the development of the numerical modeling approach described here.

### 3.1. Moving Flap

**Figure 1:**
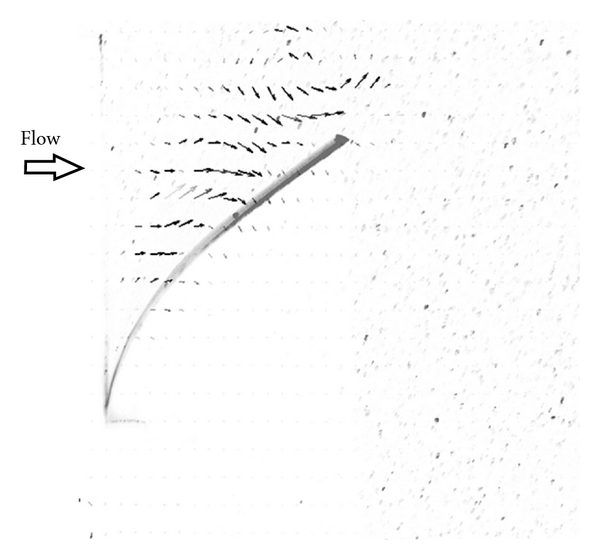
Image from the flap displacement PIV experiment. The flap (grey) is displaced by a jet of fluid, and the flow velocity is determined using PIV. The optical PIV particles appear as speckles, and the velocities are shown using arrows.

The experimental apparatus consisted of a cellulose acetate flap (taken from an overhead transparency) that was fixed on one end and placed in a 15.4 cm cube filled with water and contrast agent particles (Figure 1). A centrifugal pump was used to generate a jet of water with a diameter of 2.2 cm for a duration of 200 ms. Additional details regarding the experimental setup can be found in [33]. For the numerical simulation, the experimental system was nondimensionalized and the simulation was run using a Reynolds number of 1000 based on the inlet tube diameter, which is small enough to ensure that no turbulent effects are present.

**Figure 2:**
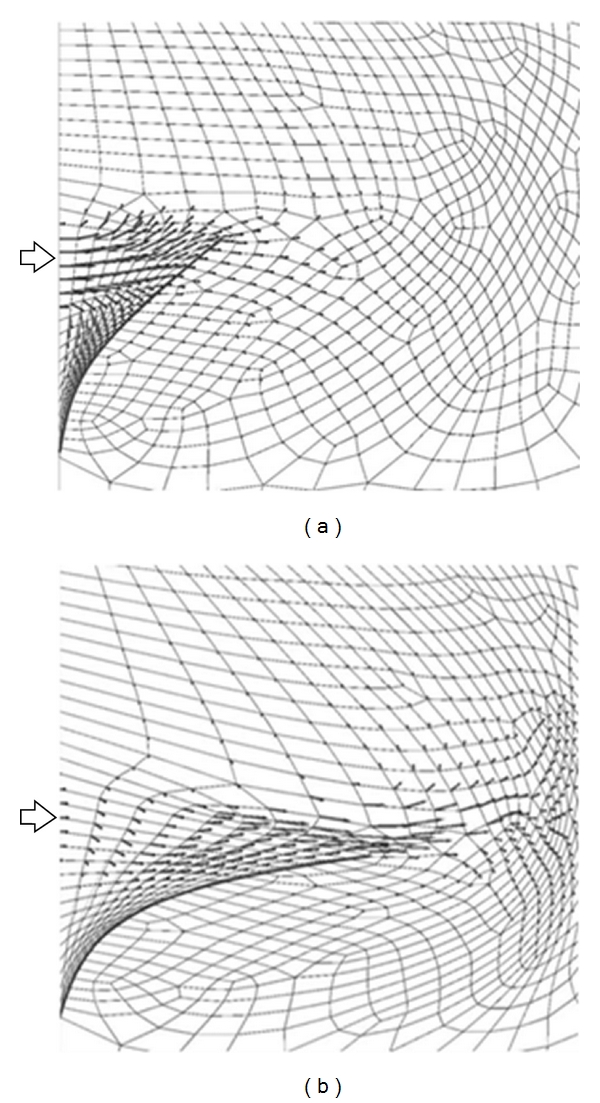
WLSFEM simulation of the moving flap experiment at (a) 0.0 sec and (b) 0.2 sec. The finite element grid deforms in response to the moving flap, and the PIV data is assimilated into the simulation with a boundary functional weight of 1.0.

A sample image from the moving flap experiment is shown in Figure 1, and the velocity data obtained from PIV data analysis is overlaid on the image. Because the experiment used an optical PIV technique, the seeding particles can be seen in the fluid. To simulate this model experiment, a cubic domain was meshed using hexahedral finite elements, and the flap in the no-flow or rest position was defined by a 2-dimensional surface within the cubic volume. The location of the flap at various time points during the experiment could be determined from the experimental images that were used for PIV analysis. The flap in each image was interpolated with a 5th order polynomial, and these polynomials were used to specify the displacement of the flap surface in the WLSFEM simulation at each time step. As the flap surface moved, the pseudosolid domain mapping technique was used to deform the finite element mesh in response to the flap motion (Figure 2). To clarify, the simulation did not model the solid flap because experimental data was available for specifying the flap location, but the simulation did include the impact of the flap on the fluid through the boundary conditions on the fluid (i.e., the no-slip boundary condition on the flap surface). The other boundary conditions used in the simulation were no-slip boundary conditions on the walls of the cubic domain except for the right surface of the domain, which was set to a natural boundary condition because the actual experimental system allowed outflow along this surface. The inlet velocity was set to a paraboloid with a total flow rate equal to the experimentally measured flow rate.

The WLSFEM algorithm was based on implicit time stepping, so from a numerical stability standpoint, any time step size could be used in the simulation. Here we used the same time step size in the simulation as was available from the PIV data, 20 msec. This means that PIV data was available at every time point in the simulation. If a simulation uses more time steps than are available from PIV data, then some simulation time steps cannot use assimilated PIV data or they must use interpolated PIV data. Based on error estimated by the experimentalists and the PIV software, a boundary functional weight of *w*_PIV_ = 1.0 was used for most simulations. This implies that the PIV data had a standard error similar to that of the flap displacement rate estimate and inflow velocity estimates. Figure 2 shows the simulation prediction for the velocity along a single plane in the 3-dimensional domain at two different time points. Specifically, the visualized plane is the same plane as the PIV data plane (recall the PIV data is typically restricted to a single plane) so that the differences between the PIV data and simulation prediction can clearly be seen. The simulation prediction of the velocity field is similar to the original PIV data, but it also contained less high-frequency variation (i.e., the simulation with the PIV data assimilated gave a smoother velocity field).

**Figure 3:**
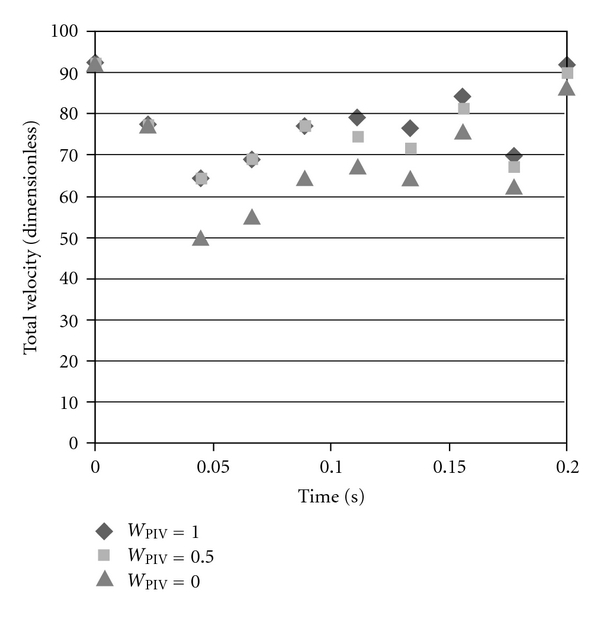
The sum of the L^2^-norm of every velocity vector along the PIV plane in the simulation versus the weight on the PIV data term in the functional. Slightly lower velocities are predicted by the simulation if the PIV data are not assimilated (*w*_PIV_ = 0.0), and including the PIV data gives slightly higher velocities. These higher velocities are partially due to high-frequency noise in the PIV data.

It is difficult to quantitatively compare the simulation predictions with and without PIV data included, but one measure is to calculate the magnitude of the velocity at every node along a surface (in this case the PIV surface) and sum those magnitudes.  Figure 3 shows this “total velocity” at every time step for different values of *w*_PIV_. A high velocity burst is seen at the beginning when the pump is turned on, then the total velocity decreases until the jet has expanded into larger parts of the domain. When *w*_PIV_ = 0.0, no PIV data is included in the simulation, and the total velocity tends to be the lowest at every time step. As *w*_PIV_ is increased, the total velocity along the PIV plane appears to converge to slightly higher values. The reason for the increase in total velocity as the boundary data weight is increased is that more high-frequency variation from the PIV data appears in the simulation result. If this same comparison is made for planes other than the PIV plane, the results are qualitatively similar, but the difference between the velocity with and without PIV data is dampened the further the plane is from the PIV plane. This is clearly a result of the fact that the further one is from the PIV plane, the less the velocity is influenced by the PIV data. 

### 3.2. Left Ventricle

**Figure 4:**
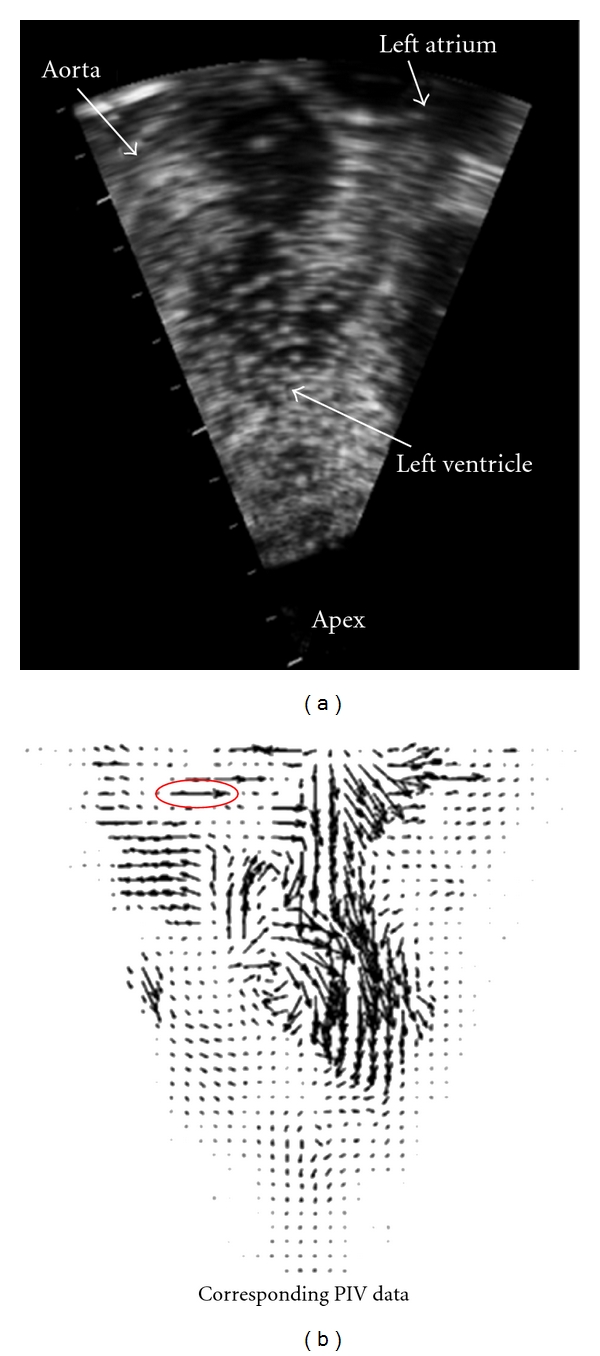
An ultrasound image showing microbubbles inside the left ventricle of the heart of a pig (left). The ultrasound probe is on the external side of the heart wall near the apex. The PIV data corresponding to the bubble motion is shown on the right. The data clearly contains some errors (e.g., the circled vector).

The PIV data for the left ventricle simulation was obtained in previous studies using an open-chest pig [4, 34, 35]. The PIV data was obtained at a higher temporal resolution (approximately 60 Hz) than the simulation time step size (50 msec.), so only a subset of the PIV data corresponding to the simulation time steps was used. A typical ultrasound image from the experiment is shown in Figure 4, and this image was obtained in the late diastole phase (i.e., near the end of the filling phase). The microbubbles in the blood appear as white spots in this image, and the bubbles appear much larger than their actual dimensions due to scattering. The ultrasound probe is placed epicardially near the apex of the heart, and most of the left ventricle is visible within the scanned region. The lack of data from outside the scanned region is not a concern because the WLSFEM can incorporate whatever data are available, and it does not have a minimum quantity of data requirement. The velocity data obtained from PIV analysis of the bubble motion are shown in Figure 4(b). The inflow from the left atrium is visible in the upper part of the domain, and a vortex can be seen near the center of the domain. During systole, the blood is ejected through the outflow tract and aorta, partially captured in the upper left of the image domain.

The WLSFEM simulation of the left ventricle required that the location of the heart walls be specified. The left ventricle was assumed to have a half-ellipsoid geometry, which is a common geometric approximation [36–38], and the motion of the heart walls was based on the measured ejection fraction of the heart and the motion observed in the ultrasound scans. The motion of the walls in the simulation is somewhat distorted by the fact that the upper surface was not allowed to move so that the cross-sectional area of the inlet (mitral valve) and outlet (aortic valve) could be kept constant. This restriction could be relaxed in the future as more experimental data becomes available. The blood velocity along the inlet was specified based on the PIV data, and the outlet flow rate was not specified (a natural boundary condition was set on the outlet). Along the heart walls, the velocity was set using a no-slip boundary condition (i.e., the fluid velocity was set equal to the wall displacement rate). The simulation was based on the dimensionless Navier-Stokes equation, and the Reynolds number was set to 1000 based on data from the pig experiments.

**Figure 5:**
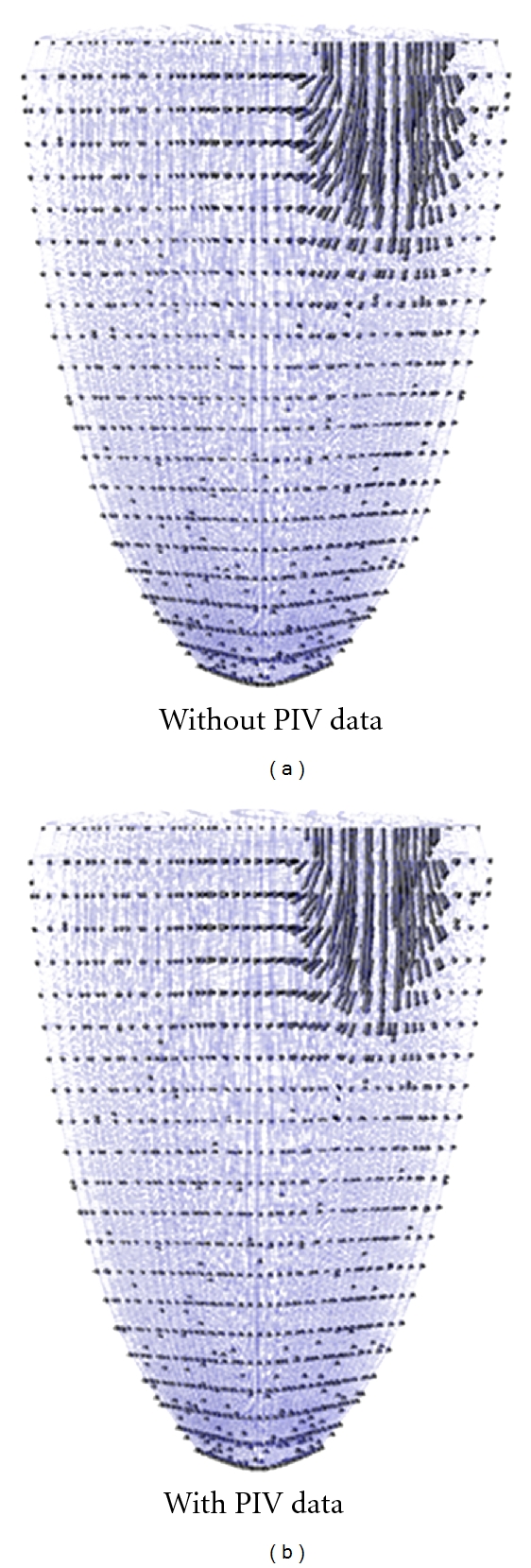
WLSFEM simulations of blood flow in the left ventricle at *t* = 0.1 sec (early diastole in our model). The left figure is the simulation without PIV data (*w*_PIV_ = 0.0), and the right figure incorporates the PIV data (*w*_PIV_ = 2.0). The PIV data has very little impact on the numerical simulation.

The simulation begins at the start of diastole, the filling of the ventricle, and the velocity along a single plane (the PIV plane) during early diastole is shown in Figure 5. The heart walls have only moved a small amount at this point in the simulation, and most of the blood flow is still near the inlet located on the right side of the upper surface. Simulations were run with *w*_PIV_ = 0.0 and *w*_PIV_ = 2.0 to explore the differences between including the PIV data and not including the data. The weight of 2.0 implies that the PIV data is actually more accurate than the velocity data along the walls, which is inaccurate because the wall location is not accurately known, and the inlet, which is also based on PIV data but is farther from the ultrasound probe. For the early time point shown in Figure 5, there are only small differences in the simulation when the PIV data are included or not included. It appears that the PIV data may have smaller velocities away from the inlet, but these differences are still very small.

**Figure 6:**
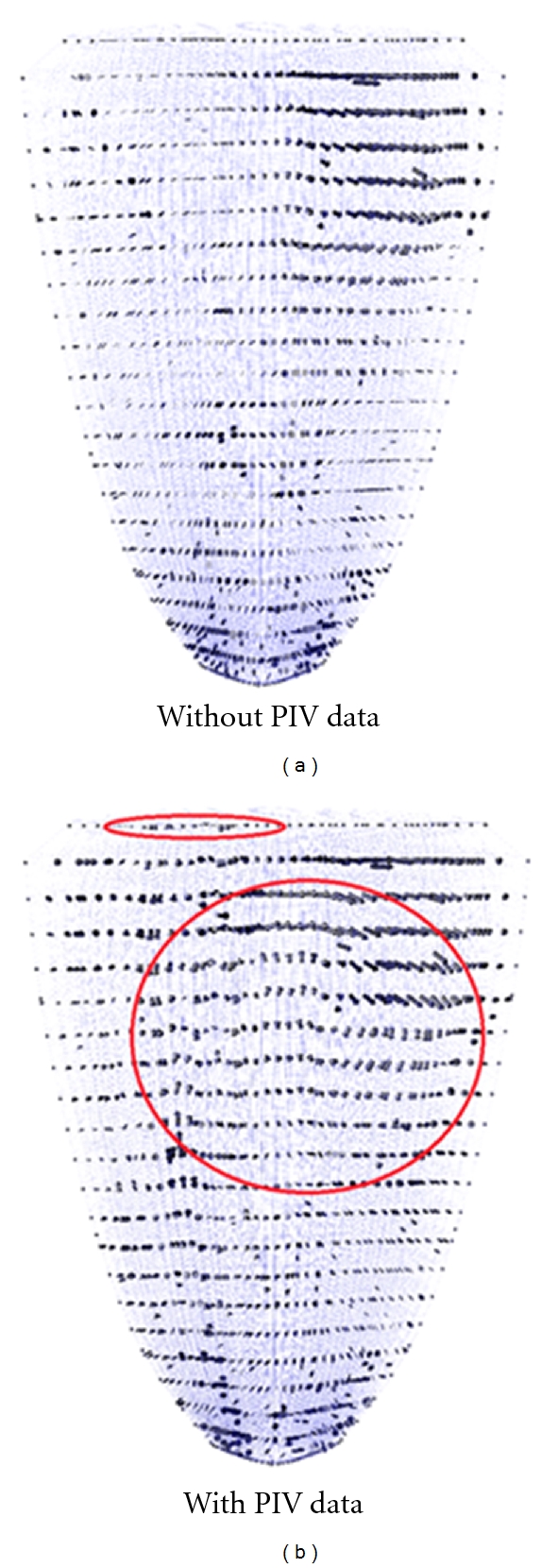
WLSFEM simulation of flow in the left ventricle at *t* = 0.5 sec (late diastole in our model). The velocity in the left figure (a) is not impacted by the PIV data, but the figure on the right includes assimilated PIV data (*w*_PIV_ = 2.0). The PIV data have a clear impact on the simulation and allow it to capture physics that may not be modeled correctly without the PIV data. For example, the simulation does not capture the effects of the mitral valve.

The PIV data has a larger impact on the simulation at later time points.  Figure 6 shows the simulation results during late diastole when the left ventricle has stopped filling. In the simulation without PIV data, Figure 6(a), there is a very weak vortex in the upper center of the ventricle, but the velocities are relatively slow in general. This vortex has been identified as potentially important for the efficient pumping of the heart [4, 39]. When PIV data are assimilated into the simulation (Figure 6(b) with *w*_PIV_ = 2.0), we see a stronger vortex and more chaotic flows in general. It appears that something may be missing from the pure numerical model (no PIV data) that is causing a higher velocity vortex. There are a number of possible reasons for this, but we will only discuss what we believe are the three most likely explanations. First, the simulation does not include the mitral valve, and the drag force applied to the blood by the valve flaps may cause a stronger vortex than would be observed without the valve. This is a very interesting result because it suggests that by properly incorporating experimental data into a simulation, we can potentially capture effects (like valves or body motion) that are often neglected in simplified numerical models. A second possibility is that the estimated Reynolds number is low and the simulation should have been run at a higher Reynolds number with greater inertial forces. Simulations run with a Reynolds number of 2000 and no PIV data showed a slightly stronger vortex than Figure 6(a), but it was still not as strong as the figure with PIV data, and it is impossible to justify a doubling of the Reynolds number estimate. A third possibility is that the idealized geometry of the left ventricle (a half-ellipsoid) resulted in a somewhat inaccurate flow field at later time points.

## 4. Conclusions

The challenge of assimilating experimental data into a computational simulation is very widespread. The general problem of interest here is solving the Navier-Stokes equations on a moving domain with additional experimental data provided by PIV experiments. In particular, the goal is the simulation of blood flow in the left ventricle with the assimilation and inclusion of 2-dimensional echo PIV data obtained using microbubbles. The WLSFEM used here is particularly well suited for this assimilation of data problem because of the flexibility in incorporating experimental data that are weighted based on accuracy. Accurate data can be closely matched with the simulation result, and less accurate data is not closely matched. The WLSFEM approach is demonstrated on two different test problems: (1) a flap that is displaced by a jet of fluid, and (2) blood flow in the left ventricle of the pig. By applying different weights to the PIV data, one can observe quantitative differences between the simulation without PIV data and the simulation that matches the PIV data more or less accurately. These comparisons can reveal inaccuracies in the model such as inaccurate boundary conditions or missing physics. The incorporation of PIV data can assist the simulation by capturing some effects that are not directly modeled. For example, in the left ventricle model presented here, the mitral valve was not modeled directly, but the effects of the valve on the blood flow could be partially captured by the simulation through the incorporated PIV data. There are other methods for incorporating experimental data into a numerical simulation, but the WLSFEM method is a flexible and efficient option for this class of problems.
